# Development of Cemented Paste Backfill with Superfine Tailings: Fluidity, Mechanical Properties, and Microstructure Characteristics

**DOI:** 10.3390/ma16051951

**Published:** 2023-02-27

**Authors:** Yafei Hu, Keqing Li, Bo Zhang, Bin Han

**Affiliations:** 1 School of Civil and Resource Engineering, University of Science and Technology Beijing, Beijing 100083, China; 2 Key Laboratory of Ministry of Education of China for Efficient Mining and Safety of Metal Mines, University of Science and Technology Beijing, Beijing 100083, China

**Keywords:** alpine mines, superfine tailings, settling properties, cemented paste backfill, fluidity, mechanical properties, microstructure, porosity

## Abstract

Previous studies have shown that the effectiveness of superfine tailings cemented paste backfill (SCPB) is influenced by multiple factors. To optimize the filling effect of superfine tailings, the effects of different factors on the fluidity, mechanical properties, and microstructure of SCPB were investigated. Before configuring the SCPB, the effect of cyclone operating parameters on the concentration and yield of superfine tailings was first investigated and the optimal cyclone operating parameters were obtained. The settling characteristics of superfine tailings under the optimum cyclone parameters were further analyzed, and the effect of the flocculant on its settling characteristics was shown in the block selection. Then the SCPB was prepared using cement and superfine tailings, and a series of experiments were carried out to investigate its working characteristics. The flow test results showed that the slump and slump flow of SCPB slurry decreased with increasing mass concentration, which was mainly because the higher the mass concentration, the higher the viscosity and yield stress of the slurry, and thus the worse its fluidity. The strength test results showed that the strength of SCPB was mainly affected by the curing temperature, curing time, mass concentration, and cement-sand ratio, among which the curing temperature had the most significant effect on the strength. The microscopic analysis of the block selection showed the mechanism of the effect of the curing temperature on the strength of SCPB, i.e., the curing temperature mainly affected the strength of SCPB by affecting the hydration reaction rate of SCPB. The slow hydration process of SCPB in a low temperature environment leads to fewer hydration products and a loose structure, which is the fundamental reason for the strength reduction of SCPB. The results of the study have some guiding significance for the efficient application of SCPB in alpine mines.

## 1. Introduction

Tailings are solid mineral wastes discharged from mineral processing plants and possess one of the highest production and lowest comprehensive utilization rates of solid waste in China [1,2,3]. Cemented paste backfill technology can fill underground mining areas and prevent surface subsidence, greatly improving the safety of mining operations and the recovery of minerals [4]. With the increasing production of tailings, and to reduce the volume of tailing stockpiles, scientists have proposed the use of tailings instead of conventional aggregates for filling to reduce the load on the environment [5,6,7]. Today, the use of solid waste fillings such as tailings has become the main development direction of filling mining technology [8]. With the gradual maturity of related technologies, the utilization rate of tailings has gradually increased. However, according to statistics, the utilization rate of tailings is still less than 40%. Tailings (especially gold tailings) contain a large number of valuable components, and the direct use of tailings to fill underground mining areas would lead to a serious waste of resources [9,10]. To improve the metal recovery rate, beneficiation technology has made great developments in recent years [11,12,13]. The ever-improving beneficiation technology has resulted in finer and finer particle sizes of the flotation tailings, as well as a significant reduction in the useful components of the tailings [14,15]. The use of such tailings for filling not only reduces the environmental load but is also beneficial to the sustainable development of the mine.

To improve the effect of superfine tailings cemented paste backfill (SCPB), scholars conducted a lot of research work. References [16,17] resorted to additional grinding of processing tailings to improve the backfill’s transportability characteristics (fluidity) and increase the strength characteristics of the created mass after hardening. This proves that, in some cases, regrinding is necessary to improve the rheological characteristics of the mixture and the strength parameters of the mass after curing. Reference [18] also showed that the use of fine fractions of tailings of water-soluble ores in backfill improves the rheological properties of backfill and increases the range of its transportation. The research showed that the CPB mix proportion has a significant effect on the performance of SCPB. Reference [19] investigated the effect of binders on the performance of SCPB to improve the utilization of ultrafine tailings and obtained the optimal binder type and dosage. Reference [20] used a highly efficient water-reducing agent to optimize the fluidity of SCPB, which greatly reduced the yield stress and viscosity of the slurry and improved the permeability of SCPB. Reference [21] showed that water loss leads to the shrinkage of the hydration products of SCPB, which in turn leads to a decrease in its strength. Reference [22] found that doped fibers in SCPB significantly improved its tensile properties and residual load carrying capacity, while improving the durability of SCPB. Reference [23] developed a new binder to replace cement, and the results showed that the calcined quarry dust and NaOH in the new binder could activate blast furnace slag and greatly improve the strength of SCPB. Reference [24] showed that SCPB has excellent fluidity and mechanical properties when the cement-sand ratio is 1:10 and the slurry mass concentration is 70%.

Studies showed that curing conditions (curing temperature and curing time, etc.) also had an effect on the properties of SCPB [25,26,27]. The curing temperature changes the microstructure and mechanical properties of SCPB [28]. At lower curing temperatures, the hydration reaction in SCPB is slow, resulting in low production of hydration products, and thus the strength of SCPB is low. A high curing temperature leads to water loss and the evaporation of water for the hydration reaction, which hinders the hydration reaction and leads to damage to the strength of SCPB. The strength of SCPB can be greatly improved only at a suitable curing temperature [29,30]. In general, the strength of SCPB is relatively low in the early stages of curing because the hydration reaction has just started. With the extension of the curing time, the material in SCPB is fully hydrated, so its strength is greatly improved. However, with the gradual end of the hydration reaction, the strength of SCPB stops growing when the curing time continues to be prolonged [31,32,33].

It can be noted from the above analysis that the study of the working characteristics of superfine tailings cemented paste backfill is a very hot topic. Therefore, the objective of this study is to comprehensively analyze the effects of different factors on the working characteristics of superfine tailings filling bodies to improve the application of superfine tailings filling bodies (especially in alpine mines). To achieve this research goal, the following issues need to be addressed: (1) the effect law of cyclone working parameters on the yield and concentration of superfine tailings slurry; (2) the effect of different factors on the fluidity of SCPB slurry; (3) the development trend of SCPB strength under the action of multiple factors; (4) the mechanism of temperature effects on SCPB strength. After conducting a series of experiments, we finally achieved the research objectives.

## 2. Materials and Methods

### 2.1. Materials

The experimental materials are mainly PO42.5 portland cement, tailings, and mixing water. The cement is the binder and the tailings are the aggregates.

#### 2.1.1. Physical and Chemical Properties of Tailings

**Figure 1:**
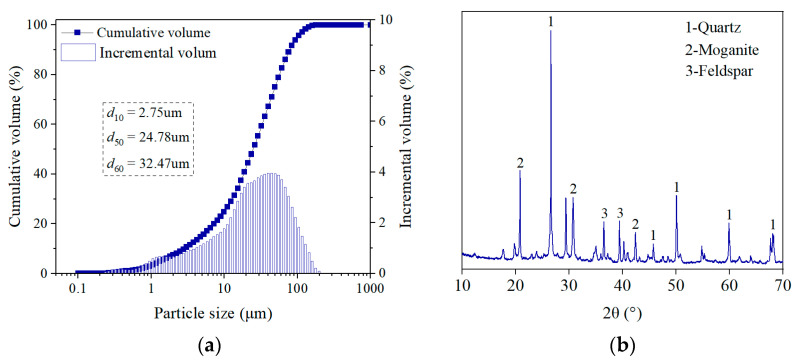
Physical and chemical properties of tailings: (**a**) Particle size; (**b**) XRD.

The tailings are taken from a gold mine in northeastern China, and its density is 2.69 g/cm^3^ when measured by the density bottle method. The particle size composition of the tailings is shown in Figure 1a, and the chemical composition is shown in Figure 1b and Table 1. The medium particle size (d_50_) of the tailings is 24.78 μm, and the percentages of particle size less than 20 μm and 75 μm are 44.15% and 90.01%, respectively, which indicate that the tailings are superfine. The tailings’ uniformity coefficient (Cu, Cu = d60/d10) is 10.81 > 10.00, indicating a better gradation. The basicity coefficient ((CaO% + MgO%)/(SiO_2_% + Al_2_O_3_%)) of tailings is 0.04 < 1, indicating that it is an acidic tailing.

The equipment model used for XRD is a Bruker D8 Focus Bragg-Brentano diffractometer, and the X-ray analysis database is the International Diffraction Data Center PDF-4+ 2010. As shown by XRD analysis, the minerals in the tailings are mainly quartz, plagioclase, and feldspar. The chemical components of the tailings are mainly SiO_2_ and Al_2_O_3_, and the proportion of the two reaches 83.10%.

#### 2.1.2. Classified Characteristics of Tailings

**Figure 2:**
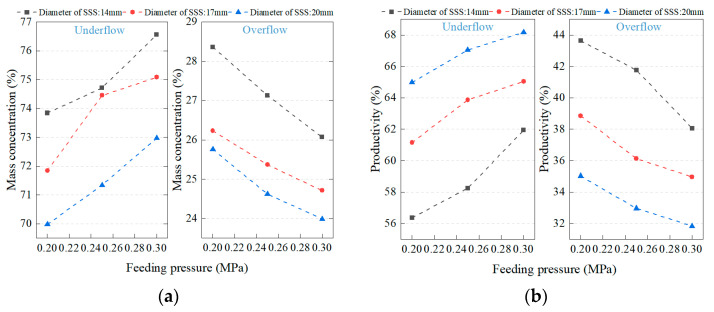
Classification characteristics of tailings (**a**) Mass concentration; (**b**) Productivity.

Since the mass concentration of the unclassified tailings slurry is too low, resulting in the low strength of the filling slurry prepared from it, the unclassified tailings need to be classified to obtain classified tailings with better filling effects. A hydrocyclone is used to classify the unclassified tailings slurry. The mass concentration and productivity of the unclassified tailings are investigated by setting different diameters (14 cm, 17 cm, 20 cm) of the sand sinking spout (SSS) and feed pressures (0.20 MPa, 0.25 MPa, 0.30 MPa), and the results are shown in Figure 2. Under the same feed pressure conditions, the underflow productivity gradually increases and the underflow mass concentration gradually decreases as the diameter of the SSS increases. When the feed pressure is 0.25 MPa, the diameter of the SSS increases from 14 mm to 20 mm, the underflow mass concentration decreases from 73.15% to 70.95%, and the underflow productivity increases from 58.24% to 67.04%. When the diameter of SSS is the same, with the increase of feed pressure, the underflow productivity gradually increases, and the underflow mass concentration also increases. When the diameter of SSS is 17 mm, the feed pressure increases from 0.20 MPa to 0.30 MPa, the underflow mass concentration increases from 71.84% to 75.08%, and the underflow productivity increases from 62.47% to 65.04%. Overall, a classified tailings slurry with an underflow mass concentration of 74.45% and a productivity of 63.87% can be obtained when the diameter of the SSS is 17 mm and the feed pressure is 0.25 MPa, and the slurry index is more in line with the engineering application requirements.

#### 2.1.3. Settlement Characteristics of the Overflow of Tailings Slurry

**Figure 3:**
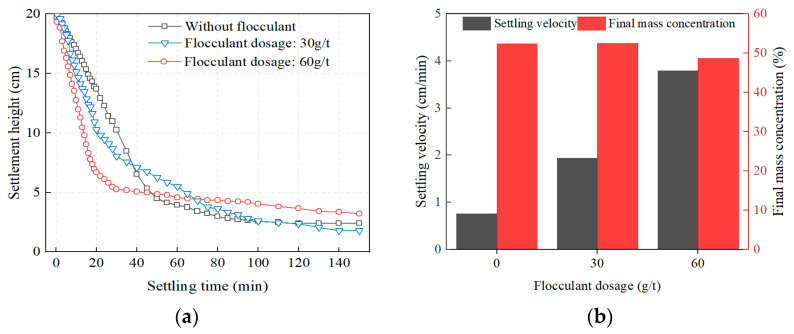
Settlement characteristics of tailings: (**a**) Settlement height; (**b**) Settling velocity.

Polyacrylamide (PAM) is used as the flocculant, and the settlement characteristics of the overflow of the tailings slurry are studied under cyclone conditions with a SSS diameter of 17 mm and a feed pressure of 0.25 MPa. The dosage of PAM is 0 g/t, 30 g/t, and 60 g/t respectively, and the corresponding experimental results are shown in Figure 3. With an increase in settlement time, the settlement height of tailings gradually increases. With an increase in PAM dosage, the settling velocity of the tailings gradually increased. The first 40 min is the tailings’ rapid settlement period, and the tailings’ settling velocity gradually slows down after more than 40 min. Under the condition of natural settlement (without flocculant), it takes 50 min for the tailings to reach the settlement inflection point. However, with the addition of a flocculant, the inflection point can be reached in 20 min. Overall, the amount of flocculant has a greater effect on the settling velocity of the tailings and a less significant effect on the final mass concentration.

### 2.2. Methods

#### 2.2.1. Strength Testing

Previous studies showed that the cement-sand ratio, slurry mass concentration, curing temperature, and curing time had significant effects on the uniaxial compressive strength (UCS) of SCPB [34,35]. To investigate the effect pattern of different factors on the UCS of SCPB, a total of 168 groups of experiments are designed to study different factors in this study, and the specific scheme is shown in Table 2. To integrate with engineering practice, the curing temperature is set to 10 °C, 15 °C, 20 °C, the cement-sand ratio is set to 1:4, 1:6, 1:8, 1:10, the slurry mass concentration is set to 60%, 65%, 70%, 73%, 76%, 79%, and the curing time is set to 3, 7, 14, 28 days.

The materials were weighed according to the proportion of each group of experiments and mixed using a JJ-5 planetary cementitious sand mixer. The mixing time was 5 min and the mixing speed was 140 r/min. The mixed slurry was poured into a triple test mold with a size of 70.7 mm × 70.7 mm × 70.7 mm and then vibrated on a ZS-15 vibrating table for 2 min to improve the compactness of the filled slurry.

#### 2.2.2. Fluidity Testing

The fluidity test mainly includes the slump test, slump flow test, and rheological properties test. The slump and slump flow are the most direct indicators of the fluidity of the slurry [36,37]. Relevant research showed that the slurry can be pumped after the slump degree exceeds 20 cm, and can satisfy the self-flow transportation requirement after it exceeds 25 cm. The main research indexes of rheological properties testing are the yield stress and viscosity of the slurry, which reflect the fluidity characteristics of the slurry itself and are the essential characterizations of the slump and slump flow. In this study, the fluidity of the tailings slurry and the filling slurry (cement-sand ratio of 1:8) with different concentrations are studied, and the research protocol is shown in Table 3.

#### 2.2.3. Microstructure Testing

Scanning electron microscopy (SEM) is the most commonly used technique to analyze the microstructure of cementitious materials, which can be used to visually analyze the microstructure of SCPB as well as the type and distribution of hydration products [38,39]. The SEM model we used in this study is ZEISS EVO1. The sample to be tested is soaked in anhydrous ethanol for more than 48 h to terminate its hydration, then dried and ground, and the microstructure of the sample to be tested is scanned with the aid of a SEM, and the results are used to analyze the microstructure and hydration products of the sample.

## 3. Results and Analysis

### 3.1. Fluidity

#### 3.1.1. Slump and Slump Flow

**Figure 4:**
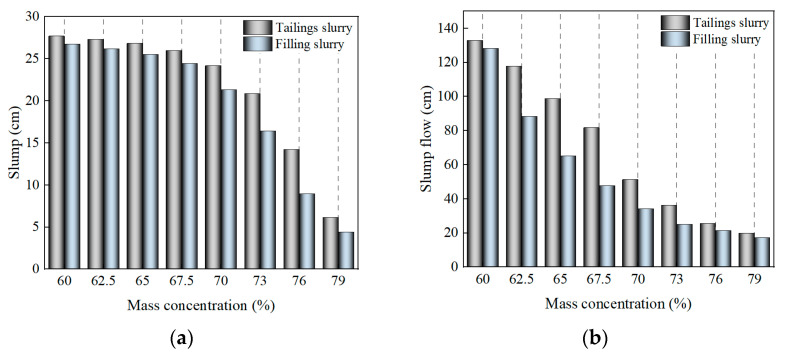
Fluidity of slurry: (**a**) Slump; (**b**) Slump flow.

Slump and slump flow is the most directly reflective index of slurry fluidity performance, and it is also an important index for mines to judge the transportability of slurry. As can be seen from Figure 4, the mass concentration has a significant effect on the slump and slump flow of the slurry, which gradually decreases with the increase of the mass concentration. This is because the free water content in the slurry gradually decreases as the mass concentration increases, leading to an increase in the flow resistance between solid particles. In addition, cement also affects the fluidity of the slurry, and the slump and slump flow are reduced after adding cement to the tailings slurry. This is because cement increases the viscosity of the slurry and increases its resistance to flow, as evidenced in Section 3.1.2.

From the change of slump, when the mass concentration of tailing slurry and filling slurry increased from 60% to 70%, the slump shows a slow decreasing trend with the increase of mass concentration; when the mass concentration of tailing slurry and filling slurry exceeds 70%, the slump shows a fast decreasing trend with the increase of concentration. The slump flow of slurry shows the opposite trend of a slump, that is, when the mass concentration of tailing slurry and filling slurry increases from 60% to 70%, the slump flow shows a rapidly decreasing trend as the mass concentration increases; when the mass concentration of tailing slurry and filling slurry exceeds 70%, the slump flow shows a slow decreasing trend as the concentration increases. The slump flow tends to decrease slowly as the concentration increases. Overall, the mass concentration of 70% is the critical concentration that affects the fluidity of the tailing slurry and the filling slurry.

#### 3.1.2. Rheological Properties

**Figure 5:**
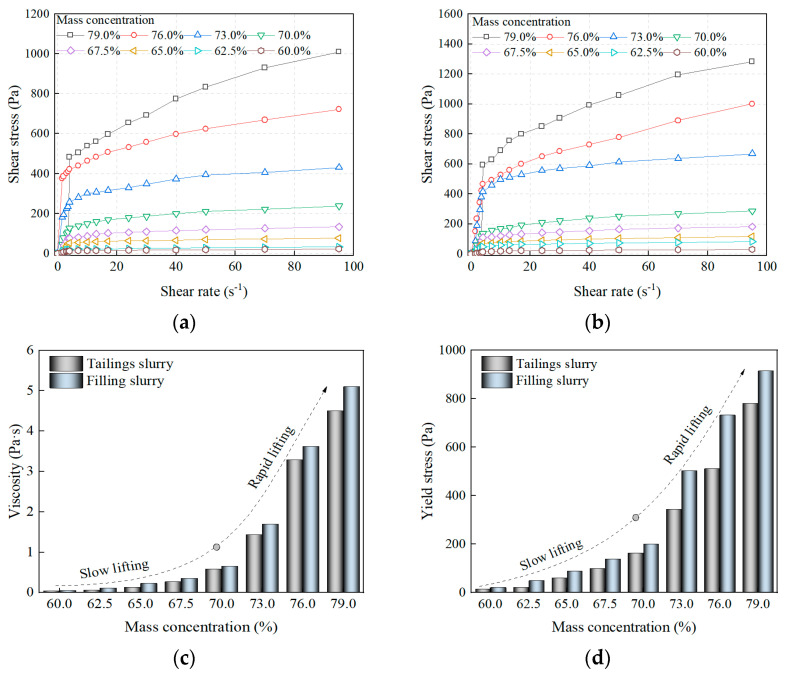
Rheological properties of slurry: (**a**) Tailings slurry; (**b**) Filling slurry; (**c**) Viscosity; (**d**) Yield stress.

Figure 5 shows the pattern of yield stress and viscosity variation with mass concentration for the tailings slurry and the filling slurry. From Figure 5a,b, the rheological properties of the slurry show an approximately linear relationship between shear rate and shear stress after the shear stress exceeds the yield stress of the slurry, which indicates that the slurry is a Bingham plastic fluid and satisfies the Bingham rheological model [40,41]. The results are shown in Table 4. The R^2^ of all the fitted curves is greater than 0.9, which indicates that the Bingham rheological model can accurately represent the rheological properties of the slurry. The Bingham rheological model is defined as follows.

(1) $$\tau =\tau_{0}+\eta \gamma$$

In Equation (1), *τ* is the shear stress, *τ*_0_ is the yield stress, *η* is the viscosity, and *γ* is the shear rate.

The yield stress can be regarded as the minimum shear stress required for the slurry to start flowing, and the higher the yield stress, the more difficult it is for the slurry to flow. From Figure 5a,b, it can be seen that as the shear rate increases, the shear stress of the tailings slurry and the filling slurry increases. At the same shear rate, the higher the slurry mass concentration, the higher the shear stress. When the mass concentration is lower than 70%, the shear stress at the same shear rate increases slightly with the increase in mass concentration. When the mass concentration exceeds 70%, the shear stress shows a significant increase, indicating that the slurry’s flow resistance is getting larger.

From Figure 5c,d, it can be seen that the mass concentration has a significant effect on the yield stress and viscosity of the slurry. With the increase in mass concentration, the yield stress and viscosity of the tailings slurry and the filling slurry show a gradually increasing trend. Meanwhile, the yield stress and viscosity of the filled slurry are larger than those of the tailings slurry, which indicates that the cement hurts the rheological properties of the slurry. The above results are consistent with the results of the slump test. When the mass concentration is below 70%, the yield stress and viscosity of the tailings slurry and the filling slurry increase slowly with the increase in mass concentration. When the mass concentration exceeds 70%, the yield stress and viscosity of the slurry increase rapidly with the increase in mass concentration. The variation of shear stress with mass concentration can be further explained based on the viscosity of the slurry. After the slurry is subjected to shear force, the internal network structure consisting of solid particles bonded together is destroyed and rearranged in the direction of shear force. The higher the concentration of the slurry, the higher its viscosity, the more difficult the network structure is to be destroyed, and thus the higher the shear stress required.

### 3.2. UCS

#### 3.2.1. Effect of the Mix Proportion on UCS

**Figure 6:**
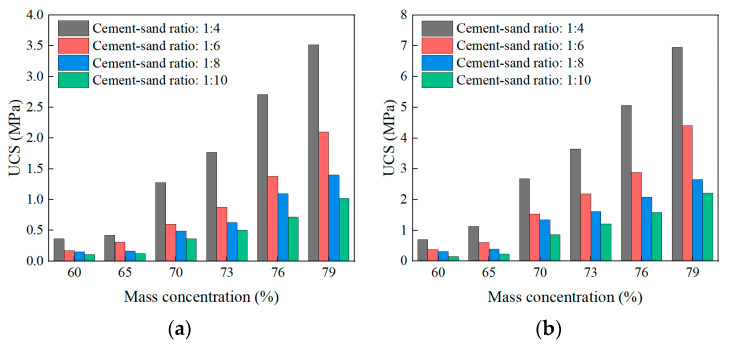

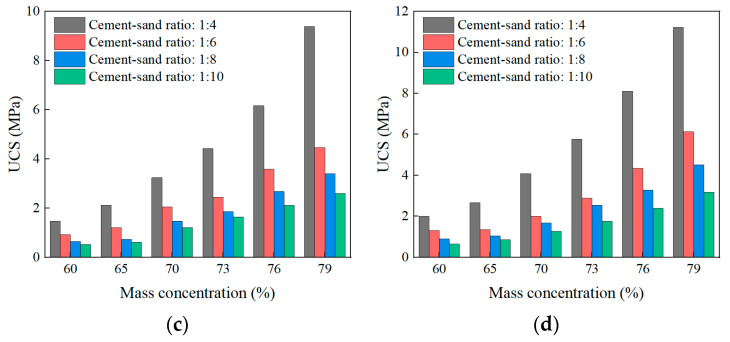
Effect of mix proportion on UCS: (**a**) 3d; (**b**) 7d; (**c**) 14d; (**d**) 28d.

The cement-sand ratio and mass concentration are the key factors to determine the mix proportion, and the UCS of SCPB under different curing times are shown in Figure 6. It can be seen from Figure 6 that the cement-sand ratio and mass concentration have a significant effect on the UCS of SCPB. When the cement-sand ratio is the same, the UCS of SCPB with different curing times tends to increase gradually with mass concentration. For example, the UCS of SCPB with a cement-sand ratio of 1:6 increased by 1127.8%, 1060.0%, 380.6%, and 367.4% for 3, 7, 14, and 28 d, respectively, when the mass concentration is increased from 60% to 79%. It can be seen that the effect of the mass concentration on the UCS is more significant the shorter the curing time is. At the same time, the enhancement effect of mass concentration on the UCS with a curing time lower than 7d is significantly better than that of the UCS with a curing time higher than 7d, indicating that the mass concentration has the most significant effect on the early UCS.

When the mass concentration is the same, the UCS of SCPB with different curing times also shows a gradual increase with the increase of the cement-sand ratio. Taking the SCPB with 70% mass concentration as an example, when the cement-sand ratio is increased from 1:10 to 1:4, the UCS increases by 252.6%, 209.9%, 169.3%, and 223.3% for 3, 7, 14, and 28 d, respectively. Overall, the degree of effect of the cement-sand ratio on UCS at different curing times is the same, while the effect of the cement-sand ratio on UCS enhancement is significantly lower than that of mass concentration. When the cement-sand ratio is higher, the UCS can also satisfy the engineering requirements at a lower mass concentration; while when the cement-sand ratio is lower, a higher mass concentration needs to be used to make the UCS of SCPB satisfy the engineering requirements.

#### 3.2.2. Effect of Curing Time on UCS

**Figure 7:**
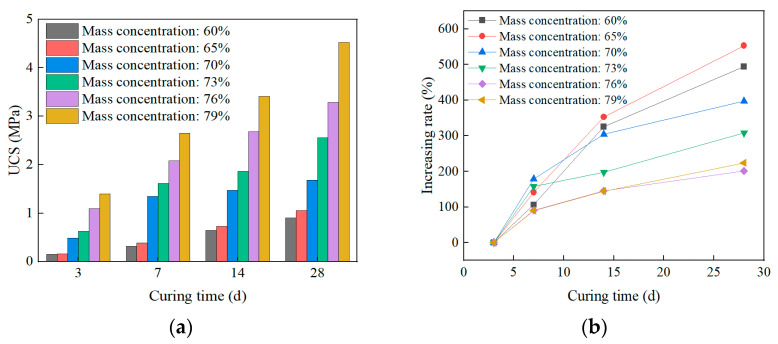
Effect of curing time on UCS: (**a**) Curing time and UCS; (**b**) Curing time and IR.

The UCS of SCPB with different curing times is shown in Figure 7. With the increase in curing time, the UCS of SCPB with different mass concentrations shows a gradually increasing trend. Under the same curing time, the UCS of SCPB gradually increases with the increase of mass concentration. The increase in curing time contributes to the full hydration reaction of cement in SCPB, thus increasing the production of hydration products. The hydration products fill the pores between the solid particles in SCPB and cement the solid particles into a three-dimensional mesh structure, thus improving the strength of SCPB, as evidenced by the microstructure analysis in Section 3.3. The higher slurry mass concentration means that its aggregate accumulation is denser and its inter-solid particle pores are less, so the UCS of high-concentration SCPB with the same curing time is higher than that of low-concentration SCPB.

The expression that defines the increase rate (IR) of the UCS of the SCPB is as follows [42,43].

(2) $$IR=\frac{F_{x}-F_{0}}{F_{0}}$$

In Equation (2), *IR* is the growth rate of UCS (%); *F_x_* is the UCS of SCPB with different curing times (MPa); *F*_0_ is the UCS of SCPB with curing 3d (MPa).

The relationship between IR and curing time is obtained by substituting UCS for different curing times in Equation (2) in Figure 7b. With the increase of the curing time, the IR of different mass concentrations shows a gradual increase, but the longer the curing time, the slower the increase of IR. When the curing time increases from 3 d to 14 d, the IR increases by 22.2%/d on average; when the curing time increases from 14 d to 28 d, the IR increases by 8.4%/d on average. This indicates that the effect of pre-mid curing on IR is significantly higher than that of late curing. In addition, the IR shows a gradual decrease with increasing mass concentration for different curing times, indicating that the effect of curing time on IR is more significant for low concentrations of SCPB.

#### 3.2.3. Effect of Curing Temperature on UCS

**Figure 8:**
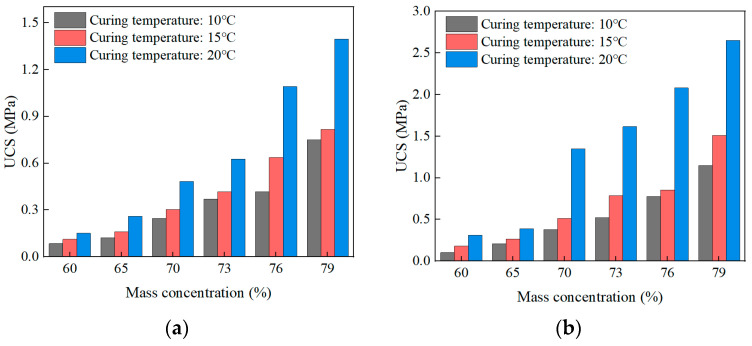

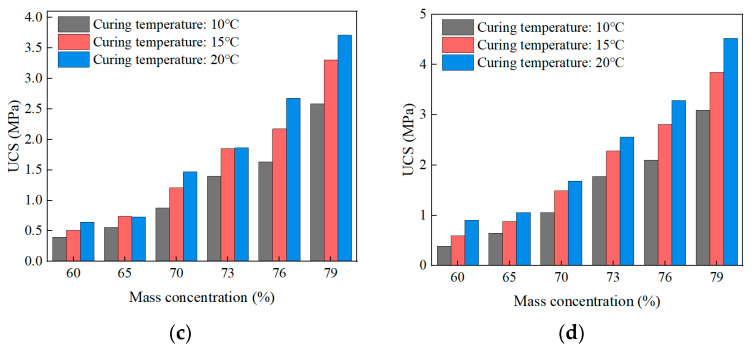
Effect of curing time on UCS: (**a**) 3d; (**b**) 7d; (**c**) 14d; (**d**) 28d.

Scientific research and engineering practice have shown that the UCS of SCPB not only grows slowly but also decreases significantly when SCPB is used in alpine mines to fill the mining area [44]. Therefore, it is important to investigate the effect of curing temperature on UCS for the engineering application of SCPB in alpine mines. The effect of curing temperature on UCS is shown in Figure 8, and the UCS of SCPB at different curing times and different mass concentrations increases with the increase of curing temperature. When the curing time is lower than 14d, the UCS of SCPB improves significantly with the increase in curing temperature; when the curing time exceeds 14d, the UCS of SCPB improves slowly with the increase of curing temperature, indicating that the effect of curing temperature on the early strength is greater than that on the middle and late strength. Meanwhile, when the curing time is lower than 14 d, the UCS of SCPB shows a substantial increase when the curing temperature is increased from 15 °C to 20 °C. In addition, the increase of the curing temperature on the strength of the filling body is more obvious after the slurry concentration exceeds 70%. Overall, the curing temperature has a large effect on the UCS of SCPB, and it is crucial to investigate the mechanism of the effect of curing temperature on the strength of the filling body.

#### 3.2.4. Correlation Analysis of Different Factors with UCS

**Figure 9:**
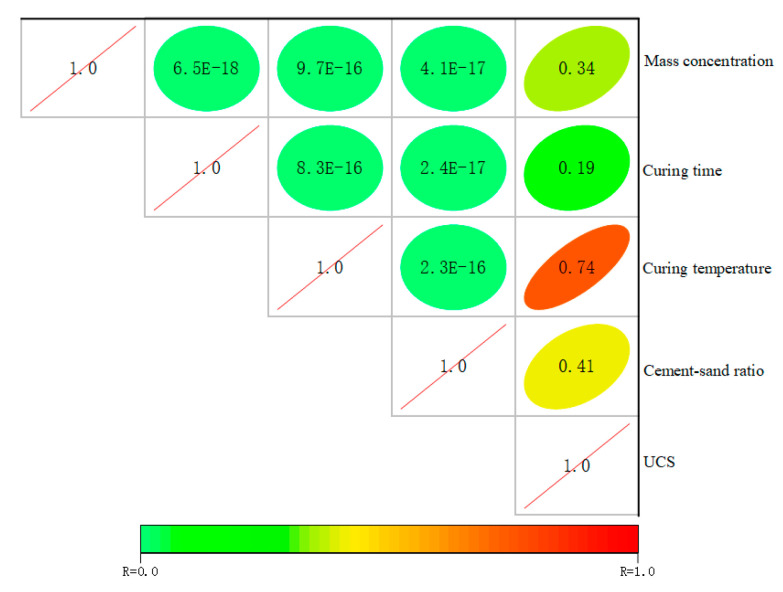
Correlation analysis between different factors and UCS.

To investigate the degree of effect of different factors on UCS, the correlation between different factors and UCS is analyzed using Pearson correlation analysis, and the results are shown in Figure 9 [45,46]. The closer the correlation coefficient *r* is to |1|, the more significant the correlation between the variables. The correlations between the four effect factors explored in this study, namely mass concentration, cement-sand ratio, curing temperature and curing time, and UCS are ranked as follows: curing temperature (0.74) > cement-sand ratio (0.41) > mass concentration (0.34) > curing time (0.19). It shows that all 4 factors are positively correlated with UCS, among which the curing temperature is strongly correlated with UCS and the curing time is weakly correlated with UCS. The results of the correlation analysis corroborated the importance of the curing temperature on the UCS of SCPB, therefore, it is important to explore the mechanism of the effect of curing temperature on the UCS of SCPB for the efficient application of SCPB in alpine regions.

### 3.3. Microstructure

#### 3.3.1. Structure of Hydration Products

**Figure 10:**
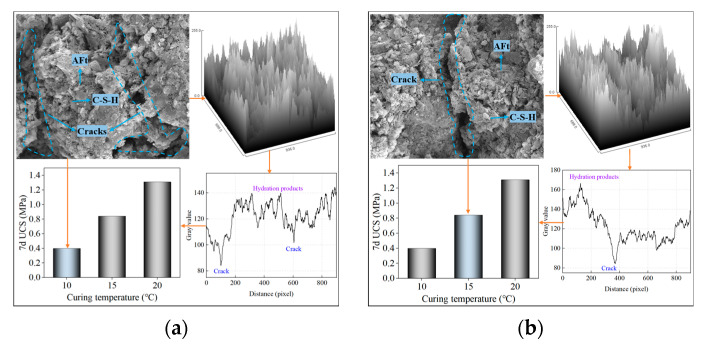

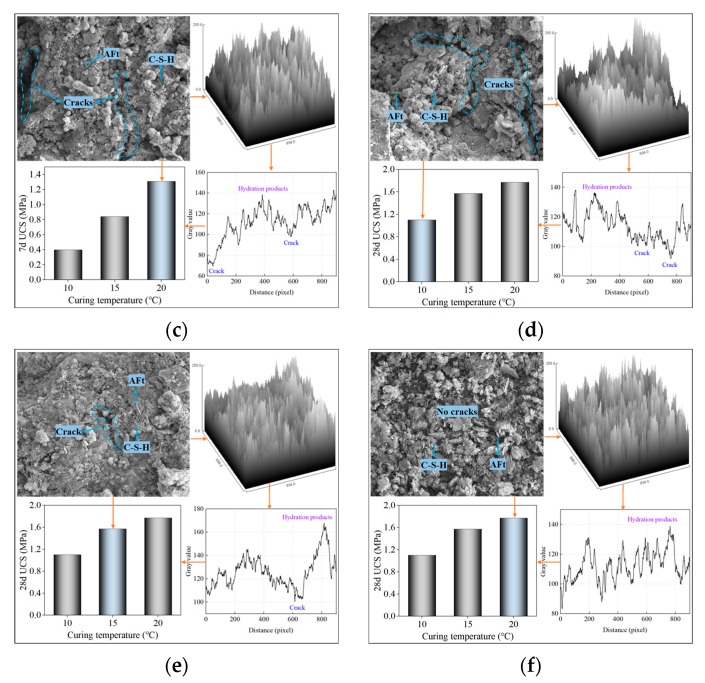
Microstructure and greyscale of different samples: (**a**) 7d, 10 °C; (**b**) 7d, 15 °C; (**c**) 7d, 20 °C; (**d**) 28d, 10 °C; (**e**) 28d, 15 °C; (**f**) 28d, 20 °C.

From the experimental results, it can be seen that SCPB has excellent fluidity and high strength when the mass concentration is 70% and the cement-sand ratio is 1:8, which can satisfy the requirements of engineering applications. The microscopic images of SCPB cured at 10 °C, 15 °C, and 20 °C for 7d and 28d are shown in Figure 10. From the SEM images, the microstructure of SCPB is mainly composed of solid particles, hydration products, and pores. The main hydration reactions occurring in SCPB are shown in Equations (3)–(5), and the main hydration reaction products are C-S-H gels, CH crystals and AFt crystals.

3(CaO·SiO_2_) + 6H_2_O = 3CaO·2SiO_2_·3H_2_O (C-S-H gel) + 3Ca(OH)_2_ (CH Crystal) (3)

2(2CaO·SiO_2_) + 4H_2_O = 3CaO·2SiO_2_·3H_2_O (C-S-H gel) + Ca(OH)_2_ (CH Crystal) (4)

3CaO·Al_2_O_3_ + 3CaSO_4_·2H_2_O + 26H_2_O = 3CaO·Al_2_O_3_·3CaSO_4_·28H_2_O (AFt Crystal) (5)

When the curing time is 7d and the curing temperature is 10 °C, a few C-S-H gels and AFt crystals are formed in SCPB with several cracks, and the structure of SCPB is relatively loose at this time. When the curing temperature is increased to 15 °C, the C-S-H and AFt in SCPB increase significantly, and the quantity of cracks is relatively reduced but still very significant. When the curing temperature reaches 20 °C, the C-S-H and AFt in SCPB increase greatly, and these hydration products fill into the cracks and make the cracks close gradually. When the curing temperature is 10 °C for 28d, the hydration products in SCPB are significantly more than those in the specimens cured for 7d under the same condition, and the cracks in SCPB are significantly reduced under the filling effect of hydration products. When the curing temperature is increased to 15 °C, the fractures in SCPB are substantially less, and its structure tends to be complete. When the curing temperature is increased to 20 °C, the hydration products in SCPB almost fill the pore structure, and its structure is more complete. The low temperature inhibits the hydration reaction of SCPB leading to a lower hydration rate, and the hydration rate is greatly increased when the temperature is increased, and a large number of products are generated to fill the solid particles so that SCPB becomes a three-dimensional cemented structure, improving its UCS.

The raised part of the two-dimensional and three-dimensional grayscale images of the microstructure of SCPB are solid particles or hydration products, and the depressed part is the pore structure [47,48]. From Figure 10a–c, it can be seen that when the curing temperature is increased from 10 °C to 20 °C, the depressed part in the grayscale image of SCPB with a curing time of 7d gradually decreases and the depression range significantly decreases, the raised part increases a lot, and the pores between each raised part are more uniform. This indicates that the hydration products in SCPB gradually increase with the increase in the curing temperature, its microstructure gradually becomes denser, and the macroscopic performance shows the increase of UCS. Figure 10d–f show the SCPB with a 28d curing event at different curing temperatures, and the trend of its grayscale image is consistent with that of 7d SCPB. At low temperatures, there are more pores and fewer hydration products in SCPB, thus its grayscale values and UCS are lower. After increasing the temperature, the hydration products in SCPB increase and the pores decrease, and the structure is dense, thus its grayscale value and UCS are higher.

#### 3.3.2. Microscopic Pore Structures

**Figure 11:**
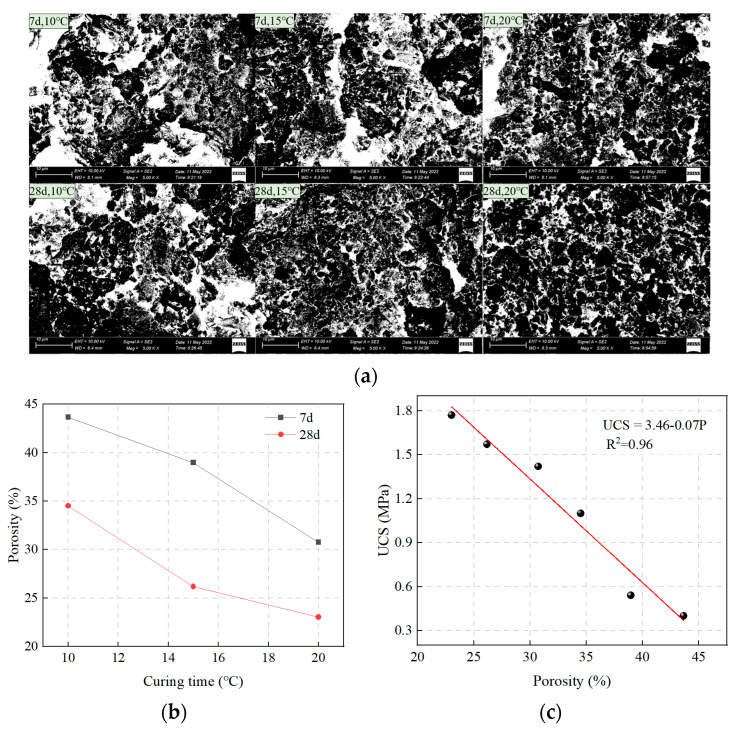
Pore structure of different samples: (**a**) Pore structure; (**b**) Curing time and porosity; (**c**) Porosity and UCS.

The pore structure of the SCPB is shown in Figure 11, where the white areas represent the pores and the black areas represent the dense solid structure. From Figure 11a, it can be seen that the percentage of the white area in SCPB gradually decreases with the increase in the curing temperature, whether the curing time is 7d or 28d, which indicates that the pores in SCPB gradually decrease. In addition, the white areas in the SCPB under low-temperature curing are widely penetrated, indicating the existence of large fractures in the SCPB. The white area in the SCPB under a high curing temperature is smaller, which indicates that the fractures have been filled with hydration products. From Figure 11b, it can be seen that the porosity of SCPB gradually decreases with the increase in the curing temperature. When the curing temperature is increased from 10 °C to 20 °C, the porosity of SCPB with curing times of 7d and 28d decreases by 29.6% and 33.3%, respectively. The linear relationship between UCS and porosity also indicates that the relationship is unique to SCPB and is not affected by factors such as curing temperature and curing time [49,50,51].

UCS = 3.46 − 0.07 · P(6)

In Equation (6), P is the porosity of SCPB (%).

## 4. Conclusions

SCPB is formulated using superfine tailings and cement, and the effects of different factors on the mechanical properties, fluidity, and microstructure of SCPB are investigated. The main conclusions are obtained as follows.

(1) The underflow productivity gradually increases and the underflow mass concentration gradually decreases as the diameter of the SSS increases; the underflow productivity gradually increases and the underflow mass concentration increases with the increase of the feed pressure.

(2) The mass concentration of 70% is the critical concentration that affects the fluidity (slump, viscosity, and yield stress) of the slurry, when the mass concentration is lower than 70%, the fluidity of the slurry changes slightly, and when the mass concentration is higher than 70%, the fluidity of the slurry changes significantly.

(3) The curing temperature, curing time, mass concentration, and cement-sand ratio are positively correlated with the UCS of SCPB, among which the curing temperature is strongly correlated with the UCS and the curing time is weakly correlated with the UCS.

(4) The low temperature inhibits the hydration reaction of SCPB, resulting in fewer hydration products. After increasing the temperature, the hydration products increase a lot and fill in between the solid particles, which constitute the cement structure and makes the structure of SCPB denser and the pores reduce a lot, thus enhancing the UCS.

## Figures and Tables

**Table 1:** Chemical element content of tailings.

Element	SiO_2_	CaO	MgO	Fe_2_O_3_	Al_2_O_3_	Na_2_O	K_2_O	SO_3_
Content (%)	69.48	5.37	3.14	2.35	13.62	0.17	0.55	0.61

**Table 2:** Experimental scheme.

Curing Time (°C)	Cement-Sand Ratio	Mass Concentration (%)	Curing Time (d)
Effect of mix proportion			
20	1:4, 1:6, 1:8, 1:10	60, 65, 70, 73, 76, 79	3, 7, 14, 28
Effect of curing time			
20	1:8	60, 65, 70, 73, 76, 79	3, 7, 14, 28
Effect of curing temperature			
10, 15, 20	1:8	60, 65, 70, 73, 76, 79	3, 7, 14, 28

**Table 3:** Experimental scheme.

Research Subjects	Cement-Sand Ratio	Mass Concentration (%)	Indicators
Tailings slurry	1:0	60, 62.5, 65, 67.5, 70, 73, 76, 79	Slump, Slump flow, and Rheological parameters
Filling slurry	1:8	60, 62.5, 65, 67.5, 70, 73, 76, 79	

**Table 4:** Rheological parameters and fitting curves of slurry.

Mass Concentration	Slurry Type	Fitting Curve	R^2^	Slurry Type	Fitting Curve	R^2^
60.0%	Tailings slurry	τ = 14.57 + 0.04γ	0.97	Fillings lurry	τ = 21.50 + 0.05γ	0.98
62.5%		τ = 22.06 + 0.06γ	0.96		τ = 63.58 + 0.11γ	0.99
65.0%		τ = 60.63 + 0.13γ	0.99		τ = 83.39 + 0.23γ	0.98
67.5%		τ = 100.33 + 0.27γ	0.97		τ = 138.21 + 0.35γ	0.97
70.0%		τ = 163.94 + 0.58γ	0.95		τ = 200.52 + 0.66γ	0.95
73.0%		τ = 344.25 + 1.61γ	0.92		τ = 553.19 + 1.70γ	0.97
76.0%		τ = 512.74 + 3.18γ	0.94		τ = 732.84 + 3.62γ	0.94
79.0%		τ = 781.38 + 4.53γ	0.91		τ = 915.07 + 5.10γ	0.93

## Data Availability

The data generated in this study are available upon request.
